# Effects of the agility boot camp with cognitive challenge (ABC-C) exercise program for Parkinson’s disease

**DOI:** 10.1038/s41531-020-00132-z

**Published:** 2020-11-02

**Authors:** Se Hee Jung, Naoya Hasegawa, Martina Mancini, Laurie A. King, Patricia Carlson-Kuhta, Katrijn Smulders, Daniel S. Peterson, Nancy Barlow, Graham Harker, Rosie Morris, Jodi Lapidus, John G. Nutt, Fay B. Horak

**Affiliations:** 1 Department of Neurology, Oregon Health & Science University, Portland, OR USA; 2 Department of Rehabilitation Medicine, Seoul Natl. Univ. Boramae Medical Center, Seoul, Republic of Korea; 3 Department of Rehabilitation Science, Faculty of Health Sciences, Hokkaido University, Sapporo, Hokkaido Japan; 4 Research Department, Sint Maartenskliniek, Nijmegen, The Netherlands; 5 Arizona State University, College of Health Solutions, Phoenix, AZ USA; 6 VA Portland Health Care System, Portland, OR USA

**Keywords:** Parkinson's disease, Rehabilitation

## Abstract

Few exercise interventions practice both gait and balance tasks with cognitive tasks to improve functional mobility in people with PD. We aimed to investigate whether the Agility Boot Camp with Cognitive Challenge (ABC-C), that simultaneously targets both mobility and cognitive function, improves dynamic balance and dual-task gait in individuals with Parkinson’s disease (PD). We used a cross-over, single-blind, randomized controlled trial to determine efficacy of the exercise intervention. Eighty-six people with idiopathic PD were randomized into either an exercise (ABC-C)-first or an active, placebo, education-first intervention and then crossed over to the other intervention. Both interventions were carried out in small groups led by a certified exercise trainer (90-min sessions, 3 times a week, for 6 weeks). Outcome measures were assessed Off levodopa at baseline and after the first and second interventions. A linear mixed-effects model tested the treatment effects on the Mini-BESTest for balance, dual-task cost on gait speed, SCOPA-COG, the UPDRS Parts II and III and the PDQ-39. Although no significant treatment effects were observed for the Mini-BESTest, SCOPA-COG or MDS-UPDRS Part III, the ABC-C intervention significantly improved the following outcomes: anticipatory postural adjustment sub-score of the Mini-BESTest (*p* = 0.004), dual-task cost on gait speed (*p* = 0.001), MDS-UPDRS Part II score (*p* = 0.01), PIGD sub-score of MDS-UPDRS Part III (*p* = 0.02), and the activities of daily living domain of the PDQ-39 (*p* = 0.003). Participants with more severe motor impairment or more severe cognitive dysfunction improved their total Mini-BESTest scores after exercise. The ABC-C exercise intervention can improve specific balance deficits, cognitive-gait interference, and perceived functional independence and quality of life, especially in participants with more severe PD, but a longer period of intervention may be required to improve global cognitive and motor function.

## Introduction

Parkinson’s disease (PD) is a neurodegenerative disorder characterized by motor and non-motor impairments. Although pharmacological and surgical interventions are the mainstay of treatment, the effect is frequently suboptimal in alleviating balance and gait impairments in PD^1,2^. Exercise is advocated as an adjunct to pharmacological therapy for balance and gait problems in PD^2,3^. However, recent studies have shown that mobility and cognition, particularly executive function and attention, are deeply interconnected and affect each other in PD^4^. For example, individuals with PD show loss of gait automaticity with increased attention to control balance and gait^5,6^. Slow speed and short strides during dual-task walking reveal loss of automaticity and functional mobility for daily living and suggests increased fall risk in people with PD^7^.

Given the evidence of cognitive network involvement in mobility tasks and overlap between cognitive and motor function^4,8^, integrated motor and cognitive training may increase functional improvement in individuals with PD^9–11^. However, few intervention protocols directly address deficits related to both gait/balance and cognitive dysfunction to improve functional mobility in people with PD^2,3^. People with PD frequently show impairment in executive function, such as set-shifting, inhibition, and updating, as well as sustained, selective, divided attention and attentional switching^11^. So, we designed a cognitively challenging mobility training that included training these aspects of cognition.

Mobility, the ability of a person to move safely in a variety of environments in order to accomplish functional tasks, requires dynamic balance control to quickly and effectively adapt locomotion, posture, and postural transitions to changing environmental and task conditions^12^. Such dynamic balance control requires complex planning of motor sequences, sensorimotor agility, ongoing evaluation of environmental cues and contexts, the ability to quickly switch motor programs when environmental conditions change, and the ability to maintain safe mobility during multiple motor and cognitive tasks. In fact, neuroplasticity is enhanced best with intensive, complex, rewarding activities. Therefore, by improving dynamic balance, we improve a critical aspect of mobility. In previous studies^13,14^, we found that people with PD in a group Agility Boot Camp program, but not people in individual therapy, or in a treadmill aerobic program, significantly improved stride velocity, gait variability, arm swing and trunk stability in gait.

The Agility Boot Camp-Cognitive Challenge (ABC-C)^11^ builds off our previously described Agility Boot Camp (ABC)^13^ program, incorporating elements to challenge both executive function/attention and systematic mobility progressions via simultaneous execution of demanding physical and cognitive tasks. This new ABC-C program was designed to add challenge to the impaired attention and executive functions known to be affected by PD (divided attention, set-switching and inhibition) and relevant to mobility disability^11^.

We hypothesized that a 6-week ABC-C program can induce functional improvements in balance, dual-task performance, and cognition in individuals with PD. We also investigated whether baseline severity of motor or cognitive impairments were predictive of efficacy of the program.

## Results

### Study flow and adverse events

**Fig. 1:**
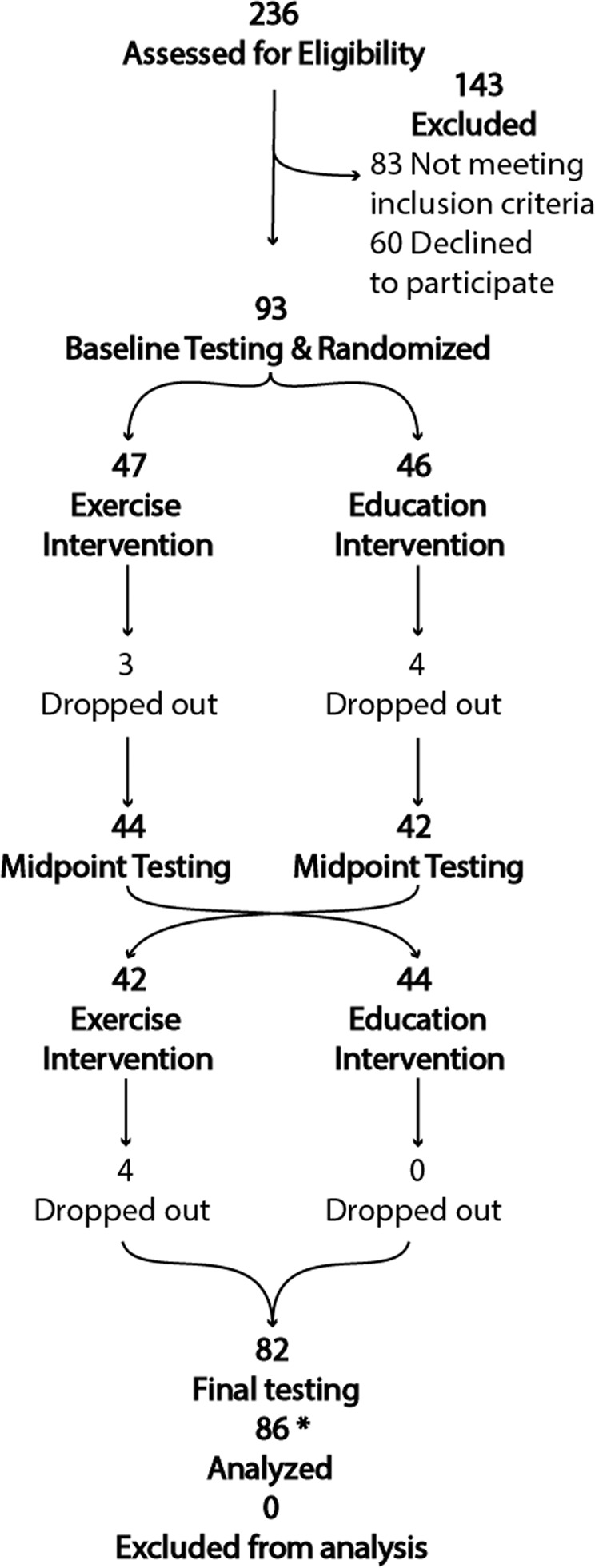
CONSORT diagram. 236 people with Parkinson’s disease were assessed for eligibility. Of these, 143 did not meet inclusion criteria, and 60 declined to participate in the study, leaving 93 subjects consented and randomized into the two intervention groups and 86 subjects’ data analyzed after dropouts. During the first intervention, 3 subjects dropped out during Exercise intervention and 4 dropped out during Education intervention. During the second intervention, 4 subjects dropped out during the Exercise intervention. One subject fell during the exercise class resulting in a hip fracture. There were also 3 minor adverse events that did not result in drop-outs: 2 fell during the exercise class and 1 fell getting out of a car.

The trial flow is summarized in the consort diagram of Fig. 1. A total of 86 participants completed at least one intervention with baseline and post-intervention assessments and were included in the analysis. The Exercise-first and Education-first groups were similar in demographic characteristics, disease duration, disease severity, mobility and cognitive function and level of physical activity at baseline (Table 1). There were no reported medication changes or major life, health or activity changes during the trial. We recently published a study on a subset of these participants^15^.

**Table 1:** Demographic data.

	All (*N* = 86)		Exercise First (*N* = 44)		Education First (*N* = 42)		*p*-Value
	Mean	*SD*	Mean	*SD*	Mean	*SD*	
Male/Female	58/28		30/14		28/14		0.881^b^
Age	68.8	7.6	67.7	6.7	70.0	8.2	0.152
Height (cm)	174.0	9.6	174.0	10.3	174.1	8.9	0.997^a^
Weight (kg)	79.4	15.3	81.5	15.6	77.2	14.7	0.195
Disease duration (yrs)	6.5	5.0	6.2	4.4	6.7	5.5	0.921^a^
MDS-UPDRS							
Total	68.2	20.4	67.2	20.2	69.3	20.7	0.651
Part II	13.8	7.2	14.5	7.4	13.1	6.8	0.386
Part III	42.3	12.2	40.7	11.1	43.9	13.1	0.232
PIGD score	5.4	2.8	4.9	2.5	5.9	3.0	0.094^a^
Hoehn & Yahr stage	1/69/8/8		1/38/4/1		0/31/4/7		0.104^b^
(I/II/III/IV)							
FoG+/FoG−	42/44		23/21		19/23		0.514^b^
NFOGQ	5.8	7.7	6.3	7.9	5.2	7.4	0.584^a^
Freezing ratio	1.2	1.6	1.1	1.0	1.4	2.0	0.272^a^
Mini-BEST							
Total	18.1	4.8	18.6	4.3	17.5	5.2	0.438^a^
APA	3.5	1.4	3.5	1.3	3.5	1.4	0.863^a^
APR	3.7	1.6	3.9	1.6	3.5	1.6	0.235^a^
SO	5.0	1.3	5.2	1.1	4.8	1.5	0.492^a^
Gait	5.8	1.8	6.0	1.8	5.7	1.7	0.292^a^
ABC scale	80.4	16.0	80.3	17.7	80.4	14.0	0.635^a^
SCOPA-COG	28.1	4.9	28.8	4.8	27.5	4.9	0.184^a^
PDQ-39							
Total	16.5	11.6	16.7	11.5	16.3	11.8	0.788^a^
Mobility	15.9	16.8	15.2	17.5	16.7	16.0	0.618^a^
ADL	19.1	15.8	21.5	16.2	16.6	15.0	0.125^a^
Exercise intensity	54/32		28/16		26/16		0.868^b^
(*N*: Light/Moderate)							

Groups compared using independent sample *t*-test, Mann–Whitney *U*-test or Chi-squared test and significance level of 0.01 (^a^Mann–Whitney *U*-test, ^b^Chi-squared test).

*PD* Parkinson’s disease, *MDS-UPDRS* Movement Disorder Society-Sponsored Revision of the Unified Parkinson’s Disease Rating Scale, *PIGD* Postural Instability and Gait Disability, *FoG* Freezing of Gait, *NFOGQ* New Freezing of Gait Questionnaire, *Mini-BEST* Mini Balance Evaluation Systems Test, *APA* Anticipatory Postural Adjustment, *APR* Automatic Postural Response, *SO* Sensory Orientation, *Gait* Dynamic Gait, *SCOPA-COG* Scales for Outcomes in Parkinson’s disease-Cognition, *PDQ-39* Parkinson’s Disease.

### Perceived exertion and compliance

There were no significant differences between Exercise-first or Education-first groups in the rate of perceived exertion for exercise and for compliance. Moderate to heavy RPE for exercise was reported in both groups (exercise-first group: 6.3 ± 0.9, education-first group: 6.2 ± 1.1 out of 10 points). Both groups showed moderately high compliance for the exercise and for the education interventions (Exercise-first group, 70.3 ± 6.2 % and 68.0 ± 18.7%; Education-first group, 66.8 ± 1.7% and 71.6 ± 13.9%). The Exercise-first group performed at a higher exercise challenging level compared to the Education-first group at the end of the study (2.75 ± 0.26 versus 2.53 ± 0.35, *p* = 0.003).

### Balance

**Fig. 2:**
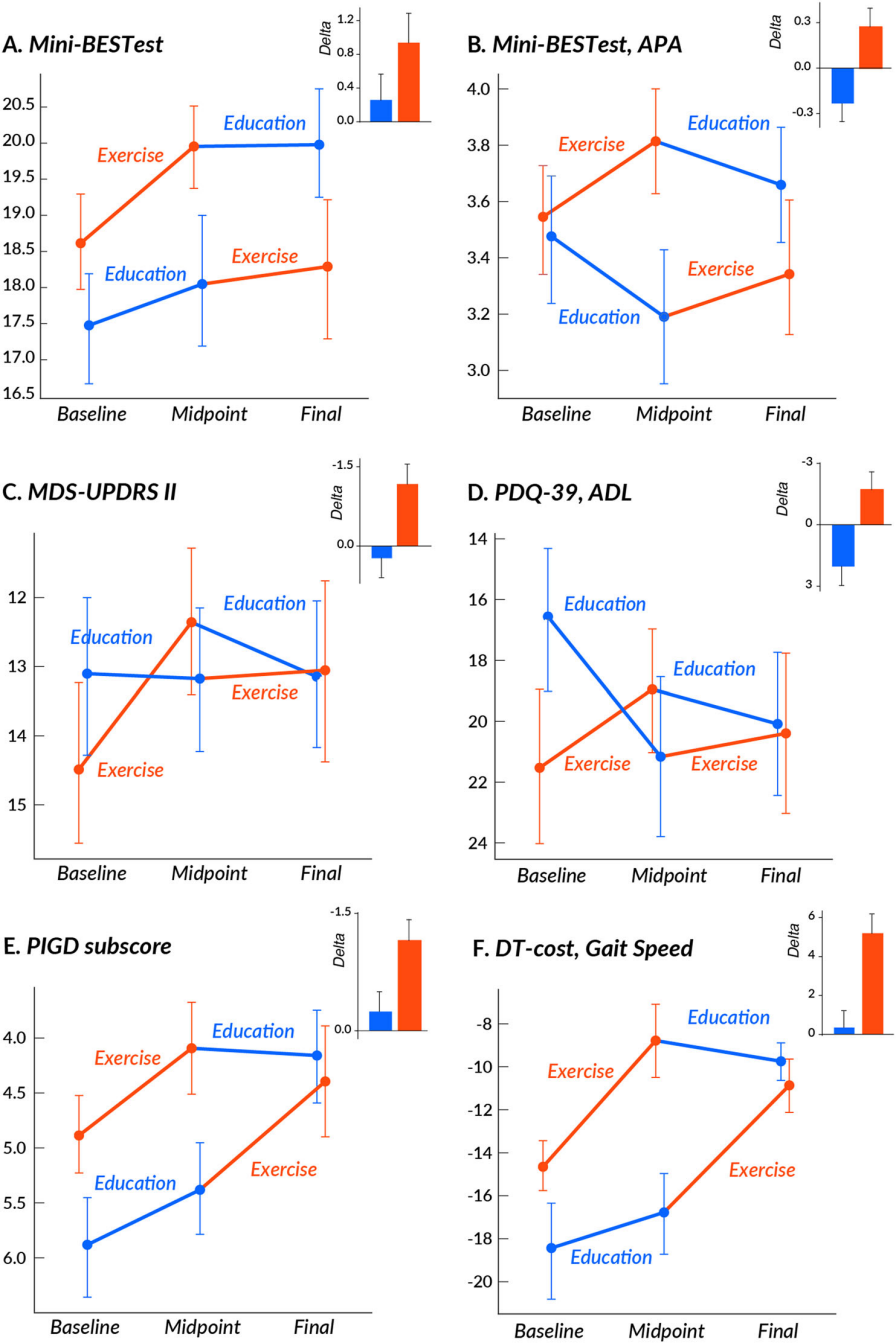
Mean and SE of outcomes at the 3 time-points in each group. The bar graphs are mean and SE of the delta after Exercise (red) and after Education (blue). **a** Mini-BESTest total score, **b** Mini-BEST APA subscore, **c** MDS-UPDRS Part II score, **d** PIGD subscore, **e** PDQ-39, ADL subscore, and **f** Dual-task cost (% change from single task) on gait speed.

**Fig. 3:**
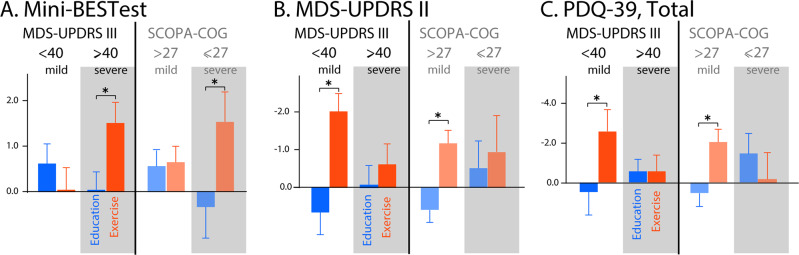
Results by disease and cognitive severity. Mean and SE of the delta after Exercise (red) and after Education (blue) when splitting subjects based on motor severity (MDS-UPDRS Part III) or cognitive severity (SCOPA-COG) for **a** Mini-BESTest total, **b** MDS-UPDRS II and **c** PDQ-39.

The Mini-BESTest total score did not have a significant treatment effect, indicating that the changes in the Mini-BESTest total score after exercise were similar to the changes in the Mini-BESTest total score after education (Fig. 2a and Table 2). However, a secondary analysis found that the APA domain of the Mini-BESTest significantly improved after exercise but not after education (*p* = 0.004, Fig. 2b). In addition, when stratifying for motor severity (based on the MDS-UPDRS III) or cognitive function (based on the SCOPA-COG), we found that only participants in a more severe motor or cognitive stage (MDS-UPDRS III > 40 or SCOPA-COG < 27) significantly improved their total Mini-BESTest scores after Exercise, but not after Education (Fig. 3a). See Supplementary Tables 1–3 for details.

**Table 2:** Mean and SE of the change in outcome measures after Exercise and Education interventions. Intervention effects of a linear mixed-effects model are reported (order and period effects are in Supplementary materials). All measures were tested in the off-levodopa state.

Clinical measures	Change after Exercise		Change after Education		Intervention effect
	Mean	SE	Mean	SE	*p*-Value
Mini-BEST					
Total	0.94	0.36	0.26	0.31	0.2
**APA**	0.27	0.13	−0.24	0.12	**0.004**
APR	0.09	0.15	0.27	0.15	0.4
SO	−0.04	0.09	0.02	0.09	0.6
Gait	0.62	0.20	0.20	0.18	0.1
Daul-Task Cost					
**DTC**_**motor**_ **gait speed (%)**	5.02	1.07	0.27	1.01	**0.001**
DTC_motor_ stride length (%)	2.83	0.82	0.84	0.96	0.1
DTC_cog_ (%)	1.66	1.81	1.22	2.79	1.0
MDS-UPDRS					
Total	−2.47	1.15	0.24	1.38	0.1
**Part II**	−1.17	0.38	0.23	0.38	**0.01**
Part III	−1.46	0.80	−0.41	0.88	0.4
**PIGD score**	−0.93	0.21	−0.20	0.21	**0.02**
PDQ-39					
Summary index	−1.41	0.68	−0.12	0.55	0.2
Mobility	−0.72	0.94	0.94	0.87	0.2
**ADL**	−1.73	0.89	2.06	0.89	**0.003**
SCOPA-COG					
	1.50	0.32	0.66	0.40	0.1

Results from a linear mixed models for the change of each clinical measure after intervention. Letters in bold indicate significant intervention effects at *p* < 0.05.

*MDS-UPDRS* Movement Disorder Society-Sponsored Revision of the Unified Parkinson’s Disease Rating Scale, *PIGD* Postural Instability and Gait Disability, *Mini-BEST* Mini Balance Evaluation Systems Test, *APA* Anticipatory Postual Adjustment, *APR* Automatic Postural Response, *SO* Sensory Orientation, *Gait* Dynamic Gait, *DTC* Dual-Task Cost, *PDQ-39* Parkinson’s Disease Questionnaire-39, *SCOPA-COG* Scales for Outcomes in Parkinson’s disease-Cognition, *APA* Anticipatory Postural Adjustment, *APR* Automatic Postural Response, *ADL* Activities of Daily Living, *DTC* Dual-Task Cost, *cog* cognition.

### Cognitive-gait interference

The DTC_motor_ on gait speed showed a significant treatment effect (*p* = 0.001, Table 2 and Fig. 2f) although the DTC_cog_ did not change. When stratifying for motor severity, participants with mild, but not severe, motor impairment (MDS-UPDRS Part III < 40) improved in both the DTC_motor_ and the DTC_cog_ (*p* = 0.04) after Exercise. When stratifying for cognitive severity, participants with worse cognitive function (SCOPA-COG score <27) improved both DTC_motor_ on gait speed (*p* < 0.001) and stride length (*p* < 0.002) after Exercise. See Supplementary Tables 1–3 for details.

### Clinical scales and independence in activities of daily living (ADL)

The PIGD sub-score of the MDS-UPDRS III significantly improved after Exercise compared to after Education (*p* = 0.02, Table 2, Fig. 2c). The total score on the MDS-UPDRS Part III did not show a significant treatment effect (*p* = 0.1, Table 2).

The MDS-UPDRS Part II score, the motor experiences of daily living, improved after Exercise but not after Education (*p* = 0.01, Table 2 and Fig. 2c). In addition, the ADL domain of the PDQ-39 showed a significant treatment effect with improvement after Exercise (*p* = 0.01) (Table 2, Fig. 2d).

When stratifying for motor severity and cognitive function, we found that only the individuals with the less severe PD motor signs (MDS-UPDRS Part III < 40) or less severe cognitive signs (SCOPA-COG > 27) significantly improved both their MDS-UPDRS Part II (*p* = 0.001 and *p* = 0.001, respectively) and their total PDQ-39 after Exercise (*p* = 0.01 and *p* = 0.01, respectively), Fig. 3c, d. See Supplementary Tables 1–3 for details.

### Cognitive function

The SCOPA-COG total score did not show a significant treatment effect (Table 2). In addition, no differences in SCOPA-COG scores were observed when the participants were stratified by the MDS-UPDRS Part III (Supplementary Table 2).

## Discussion

The ABC-C program resulted in short-term, selective improvement of balance, dual-task gait speed and independence in ADL/quality of life measures in people with idiopathic PD. However, the total Mini-BESTest did not improve with the ABC-C program, likely because the program focused broadly on mobility but not targeted to each of the four balance domains within the Mini-BESTest. Specifically, the ABC-C focused on APAs and dual-task gait, not on postural responses or sway in stance under different sensory conditions.

After 6-week of ABC-C training, improvements were observed in the APA subscore of the Mini-BESTest. Gait initiation in multiple directions and weight-shifting postural adjustments were important components of the ABC-C, such as lunging with large amplitudes and Tai Chi. This may explain why the APA subscore improved. Inadequate weight-shifting is one of the significant factors of falls in older adults with and without PD^16^. Therefore, improving APAs may be important for impacting gait initiation failure and fall risk. In fact, balance training focused on step initiation, that include APAs, has been shown effective in reducing falls among older adults in previous clinical trials^17^.

In addition, significant improvements after the ABC-C training, but not after education, were found on the dual-task cost on gait speed. This finding is consistent with the results of previous studies and showing that people with PD can improve gait performance during motor-cognitive interference situations^18–20^. Mobility in daily life frequently requires walking while performing simultaneous motor or cognitive tasks so daily life walking is more similar to dual-task, than single task walking when assessed in a laboratory environment^21^. Although control of walking should be automatic with minimal use of attentional control resources, people with PD tend to use attentional control strategies to compensate for impaired automatic motor control, resulting in reduced dual-task gait performance^22^. The improvement of dual-task cost was observed primarily for gait speed, not for stride length. Such discrepancy could be due to dual-task cost on cadence improving.

In addition, the PIGD subscore of the MDS-UPDRS, Part III, the ADL domain of the PDQ-39, and the patient-reported outcome of the MDS-UPDRS Part II all showed significant improvements after the ABC-C Exercise training, but not after Education. The PIGD score targets the severity of balance and gait dysfunction in people with PD and is associated with greater severity of non-dopaminergic symptoms^23^. PIGD severity is also a marker of advancing disease. Postural responses to the Pull test within the PIGD likely did not improve as postural responses were not practiced in the ABC-C and the postural response subcomponent of the Mini-BESTest did not improve^23^. A recent longitudinal study showed that subjects with PD face a high risk of independence loss even at early stages^24^. The ABC-C training included various dual-task conditions that resemble real-world activities and require agility and judgement, such as walking over and around obstacles while answering questions from the trainer. Therefore, getting more proficient in these challenging conditions may induce improvement in independence in activities of daily living and perceived functional independence. In addition, the results suggest that the ABC-C may change patient-reported perception of balance and activities of daily living, that are important indicators of health. In fact, clinical trials of movement impairments in neurological patients are increasingly using patient-reported measures as primary outcomes rather than clinicians’ evaluations. Ideally, measurements of patients’ mobility before and after future interventions would also use objective measures from daily life mobility to record how people actually perform.

Our findings suggest that the ABC-C training program may have differential effects depending upon individuals’ baseline severity of motor or cognitive impairments, assessed by MDS-UPDRS Part III and SCOPA-COG. In fact, only participants with mild motor impairments (MDS-UPDRS Part III < 40) significantly improved in dual-task cost for gait velocity, as well as dual-task cost on cognitive performance after the ABC-C Exercise training. However, subjects with moderate-to-severe motor impairment (MDS-UPDRS Part III > 40) did not improve in either gait or cognitive dual-task cost. No instructions were given on which task to prioritize in the dual-task condition, so it is possible that subjects with preserved cognitive function learned to allocate more attentional resources to the cognitive task, whereas subjects with mild cognitive impairment required all their attention on the gait task. The difficulty of more severely affected people with PD to improve gait-cognitive interference with practice suggests a limited ability for those with more severe PD to compensate with frontal, attentional circuits for loss of gait automaticity. However, it is not clear if gait became more automatic for those who improved dual-task cost or patients became better at switching attention quickly for dual-tasking.

In summary, although we demonstrated a potential advantage of adding cognitively challenging components to mobility agility training, there are several limitations in the current study that we need to acknowledge. First, the intervention was limited to only 6-weeks. This short duration of training might be insufficient to yield a clinically significant improvement as reported in other studies in people with PD^3,25,26^. In fact, as previously reported, people with PD often show motor learning at a slower pace and of smaller magnitude relative to age-matched individuals, therefore requiring longer training to attain and maintain motor learning^27,28^. Previous exercise studies that reported significant improvements in the Mini-BESTest total score, MDS-UPDRS, and falls generally lasted 6 months or longer^3,29–31^. Second, subjects in a group class were heterogeneous in their motor, cognitive, balance and gait deficits (including those with and without freezing of gait). Although attempts were made to customize the exercise progression level for each individual, the group of 6 per class may have limited the challenge for those with less severe impairments. Third, although subjects underwent exercise intervention in their “on” state, all outcomes were measured in their “off” state to exclude the effects of dopaminergic medication. This may have impaired fully evaluating the effects of intervention on daily life functional performance. Fourth, there was no washout period between the first and second intervention in this cross-over design, so we could not rule out a possible carry-over effects from the first interventions. However, sequence and period effects were nonsignificant. Fifth, only the immediate effects of exercise were evaluated as we did not collect data regarding long-term retention, nor falls, one of the most debilitating results of impaired mobility.

Despite these limitations, the current findings suggest important implications for clinical practice by providing evidence supporting the idea that exercise simultaneously targeted on both motor and cognitive function can enhance functional abilities in subjects with PD. Future studies should investigate the effects of a longer interventions on long-term benefits of the ABC-C, particularly in people with PD who have more severe motor and cognitive impairments.

## Methods

### Study design

The study was a cross-over, single-blind, randomized controlled trial to determine the effectiveness of the ABC-C for individuals with PD. All participants were diagnosed by movement disorders specialists as having idiopathic PD based on the United Kingdom Brain Bank criteria^32^. Participants were randomized into either an exercise-first or an education-first (active control) intervention and crossed-over after 6 weeks to receive the other intervention without a washout period. Both interventions were designed to have the same frequency with the same group and were delivered by the same certified exercise trainers, who were experienced in working with people with PD. Outcome measures were assessed at three time points: (1) baseline, (2) midpoint, after the first 6-week intervention, and (3) final, after the second six-week intervention. Independent exercise and medications were kept as stable as possible throughout the trial.

### Participants

Details are provided in the Consolidated Standards of Reporting Trials (CONSORT) flow diagram (Fig. 1). Subjects were eligible if they were: (a) 50–90 years old; (b) on stable anti-parkinsonian medication; (c) able to stand or walk for 2 min without an assistive device; and (d) able to consent to participate and to follow testing and intervention procedures. Exclusion criteria for participation were: (a) comorbidities that contraindicates exercise participation, (b) significant musculoskeletal or peripheral nervous system disorders affecting balance, (c) excessive use of alcohol or recreational drugs, (d) deep brain stimulation surgery, and (e) contraindications to MRI scans. We recently published a study on a subset (participants with Freezing of Gait) of the included participants in King, et al.^15^.

This work was approved by the joint Oregon Health & Science University (OHSU) and Veterans Affairs Portland Health Care System (VAPORHCS) institutional review board ethics committees and each participant provided written informed consent. This trial was registered on Clinicaltrials.gov (NCT02231073 and NCT02236286).

### Randomization and blinding

Subjects were randomly assigned to either the exercise-first or education-first intervention by a computerized block randomization centrally held in the Research Electronic Data Capture (REDCap) database-scheduling mode. Randomization was implemented by an independent statistician using a block size of 12 subjects (6 in each intervention). After randomization, the exercise trainer (unblinded) notified the subjects by phone. The participants were blinded to our hypothesis and expected outcomes. The researchers who performed all baseline, midpoint and final tests remained blinded to group assignment throughout the duration of the study.

### Intervention

#### Exercise

The exercises were designed as a circuit to challenge movement-skills known to be impaired in PD and included the following stations: (1) Gait training (2) PWR! Moves ©, (3) Agility course, (4) Lunges, (5) Boxing and (6) Adapted Tai Chi (See details in Appendix I in King, et al.^15^). Briefly, each station was engaged for 10–20 min with short rest periods between stations. Activity at each station was systematically progressed over 3 levels of difficulty for each participant by challenging: visual and surface conditions, restricting external sensory cues, increasing speed and resistance, by including: response inhibition or set-switching tasks, and by adding secondary cognitive tasks^9^.

The ABC-C program included 80-minute sessions including breaks, 3 times a week for 6 weeks for an overall education dose of 240 min per week. The class was a group class with 3–6 people per class and 1 to 2 research assistants to spot those participants judged by the physical therapist to have high fall risk. Both the balance and cognitive challenge levels were progressed for each station as tolerated by each individual and were recorded by the trainer. A participant was progressed when the trainer determined they were safely and accurately performing the exercise.

### Active control: education

We chose the same size group session with the same subjects and same trainer at the same location as the active control group. This control intervention was chosen to control for the socialization, group dynamics, travel and leadership effects provided by the group exercise. We intentionally selected an educational program that was useful to patients (to minimize drop-outs) but did not include education about exercise so subjects would not change their exercise habits during the control intervention. The education program was developed by our study team to be specific for people with PD and focused on self-management of care team development, sleep, nutrition, stress, mood and medication. Classes met with the same trainer for 80-minute sessions, once a week for six weeks. In order to match the dose of education intervention with exercise intervention, participants were also provided relaxation CD’s to use at home 6 times per week for 30 min at a time, for an overall education dose of 240 min per week.

The education intervention was developed to teach people how to better live with a chronic disease. It would be inappropriate in the active, control intervention, to teach subjects about exercise, balance and falls as it would likely change their exercise and fall-risk behavior/habits during the study. We previously published the interventions and protocol for this study^9^.

### Compliance

Compliance was recorded at each session by the exercise trainer for both the Exercise and Education interventions. For the education arm, participants also recorded compliance for the relaxation sessions in a logbook. The trainer coded progression of exercise difficulty at the end of each week to determine the level of exercise progression for each participant. Additionally, participants stated their rate of perceived exertion (RPE on 0–10 scale) after each exercise session.

### Assessments

All assessments were performed in the practical off levodopa state after 12-hour withdrawal of dopaminergic medication. The primary balance outcome measure was the Mini-Balance Evaluation System Test (Mini-BESTest)^33^. The Mini-BESTest^33^ is a sensitive measure of dynamic balance and includes 14 items (a maximum and best score of 28). Secondary analysis included scores of the 4 balance systems within the Mini-BESTest: Anticipatory Postural Adjustments (APA), Automatic Postural Responses (APR), Sensory Orientation (SO), and Dynamic Balance (Gait)^33^. We hypothesized that APAs and Dynamic Balance during Gait would improve the most as these were a focus of the ABC-C program.

Secondary outcome measures included measures of cognitive-gait interference, perceived mobility disability and quality of life and cognition. Cognitive-gait interference was measured with the Dual Task Cost on gait speed (DTC_motor_) calculated as the percentage change of gait speed measured without (single task, ST) and with the simultaneous cognitive task (dual-task, DT). The cognitive DTC (DTC_cog_) was calculated using the same equation considering the percentage of correct answers in reciting every other letter of the alphabet while the subjects were seated for two minutes (ST) versus while the subjects performed the 2-minute walk over a 7-meter length. In a dual-task condition, no instructions were given on which task to prioritize. Details on the protocol are published elsewhere^9,32^.

The Movement Disorders Society Unified Parkinson’s Disease Rating Scale (MDS UPDRS- Parts I-IV) was used to measure disease severity and the postural instability and gait difficulty (PIGD) subscore was calculated to assess parkinsonian gait and balance^34^. The ADL subscore of the Parkinson’s disease quality of life questionnaire (PDQ-39) was used to assess quality of life focused on mobility^35^. Cognitive assessment used the Scales for Outcome of Parkinson’s Disease-Cognition (SCOPA-COG)^36^.

A secondary analysis investigated the effect of exercise intervention based on the severity of baseline motor and cognitive impairments of subjects with separate linear mixed models. For motor severity, subjects were dichotomized with the MDS-UPDRS Part III (Part III < 40, *n* = 34, milder and Part III ≥ 40, *n* = 52, severe)^37^. For cognitive impairment, subjects were stratified based on the SCOPA-COG score (non-MCI; SCOPA-COG ≥ 27, *n* = 58 versus MCI; SCOPA-COG ≤ 27, *n* = 28)^36^.

### Statistical analysis

As this study was a crossover design, the treatment effect represents whether the change (delta) during the Exercise intervention differs from the change during Education intervention. A linear mixed-effects model was used including an indicator of treatment effect (Exercise versus Education), order effect (Exercise-first versus Education-first) and period effect (sequence of assessments) to determine whether the “difference in change” differed between Exercise and Education. In addition, the effect of Exercise and Education was expressed as the standardized response mean (SRM) for each clinical and objective measure. The SRM was calculated as the mean change between before and after each intervention period divided by the standard deviation (SD) of the change^38^. An SRM value of 0.20 represents a small, 0.50 a moderate, and 0.80 a large effect of the intervention^38^. The statistical analysis for the demographic data and clinical measures at baseline were processed using SPSS Statistics version 25.0 (IBM, Armonk, NY, USA), and a linear mixed model was calculated using MATLAB R2018b (The Mathworks Inc., Natick, MA, USA). The statistical significance was set to *p* < 0.05 for all analyses.

### Reporting summary

Further information on research design is available in the Nature Research Reporting Summary linked to this article.

## Supplementary information

Supplementary Information

Reporting Summary

## Data Availability

The data sets generated during the current study are available from the corresponding author on a reasonable request.

## Code availability

The codes generated during the current study are available from the corresponding author on a reasonable request.
