# Supplementary material for: Effects of the agility boot camp with cognitive challenge (ABC-C) exercise program for Parkinson’s disease
Source: NPJ Parkinsons Dis. 2020 Nov 2;6:31. doi: 10.1038/s41531-020-00132-z (PMC7608677; doi:10.1038/s41531-020-00132-z)
Supplement: Supplementary file 1 — Supplementary Information [file 41531_2020_132_MOESM1_ESM.pdf]

Supplementary Table 1

Linear mixed model Intervention, Order, and Period Effects. Lower and upper 95% confidence intervals (CI) for beta are also presented. Letters in bold indicate significant intervention effects at $p < 0.05$.

Clinical measures		Fixed factor	Beta	*t* value	Lower *CI*	Upper *CI*	*p* value
Mini-BEST	Total	Intervention	-0.64	-1.36	-1.56	0.29	*0.2*
		Order	-0.14	-0.31	-1.07	0.78	*0.8*
		Period	-0.78	-1.66	-1.70	0.15	*0.1*
	**APA**	Intervention	-0.51	-2.95	-0.85	-0.17	***0.004***
		Order	-0.08	-0.48	-0.42	0.26	*0.6*
		Period	0.05	0.28	-0.29	0.39	*0.8*
	APR	Intervention	0.17	0.83	-0.24	0.59	*0.4*
		Order	0.04	0.17	-0.38	0.45	*0.9*
		Period	0.19	0.90	-0.23	0.60	*0.4*
	SO	Intervention	0.08	0.60	-0.18	0.33	*0.6*
		Order	0.01	0.04	-0.25	0.26	*1.0*
		Period	-0.33	-2.57	-0.58	-0.08	*0.01*
	Gait	Intervention	-0.38	-1.46	-0.90	0.13	*0.1*
		Order	-0.10	-0.39	-0.62	0.41	*0.7*
		Period	-0.68	-2.62	-1.20	-0.17	*0.01*
Daul-Task Cost	**DTC_{motor} gait speed (%)**	Intervention	-4.77	-3.26	-7.66	-1.88	***0.001***
		Order	0.88	0.60	-2.01	3.77	*0.6*
		Period	-0.02	-0.01	-2.91	2.87	*1.0*
	DTC_{motor} stride length (%)	Intervention	-2.02	-1.61	-4.51	0.46	*0.1*
		Order	1.29	1.03	-1.19	3.78	*0.3*
		Period	-0.13	-0.10	-2.61	2.36	*0.9*
	DTC_{cog} (%)	Intervention	0.14	0.04	-6.36	6.65	*1.0*
		Order	-2.65	-0.81	-9.16	3.85	*0.4*
		Period	6.04	1.83	-0.47	12.54	*0.07*
MDS-UPDRS	Total	Intervention	2.75	1.54	-0.78	6.27	*0.1*
		Order	-1.42	-0.79	-4.95	2.11	*0.4*
		Period	-0.05	-0.03	-3.58	3.48	*1.0*
	**Part II**	Intervention	1.35	2.53	0.29	2.40	***0.01***
		Order	0.30	0.56	-0.75	1.35	*0.6*
		Period	1.01	1.90	-0.04	2.06	*0.06*
	Part III	Intervention	1.10	0.94	-1.23	3.44	*0.4*
		Order	-0.82	-0.69	-3.15	1.51	*0.5*
		Period	-0.80	-0.68	-3.13	1.53	*0.5*
	**PIGD score**	Intervention	0.73	2.46	0.14	1.31	***0.02***
		Order	-0.39	-1.33	-0.98	0.19	*0.2*
		Period	0.19	0.63	-0.40	0.77	*0.5*
PDQ-39	Summary index	Intervention	1.22	1.42	-0.48	2.93	*0.2*
		Order	0.28	0.32	-1.43	1.98	*0.7*
		Period	1.17	1.35	-0.54	2.87	*0.2*
	Mobility	Intervention	1.55	1.22	-0.96	4.06	*0.2*
		Order	0.59	0.46	-1.92	3.09	*0.6*
		Period	1.83	1.45	-0.67	4.34	*0.2*
	**ADL**	Intervention	3.78	3.03	1.32	6.25	***0.003***
		Order	1.49	1.19	-0.98	3.95	*0.2*
		Period	-0.03	-0.02	-2.49	2.44	*1.0*
SCOPA-COG		Intervention	-0.81	-1.59	-1.81	0.19	*0.1*
		Order	-0.24	-0.48	-1.24	0.76	*0.6*
		Period	-0.79	-1.56	-1.79	0.21	*0.1*

Supplementary Table 2

The change of each clinical measures after exercise and education according to the baseline severity of motor impairment

	**Mild motor impairment. (MDS-UPDRS Part III < 40)**				**Severe motor impairment (MDS-UPDRS Part III ≥ 40)**															
	Delta Exercise		Delta Education		Delta Exercise		Delta Education			*Mild motor impairment*					*Severe motor impairment*				
Clinical measures	Mean	*SE*	Mean	*SE*	Mean	*SE*	Mean	*SE*	Fixed factor	Beta	*t* value	Lower *CI*	Upper *CI*	*p* value	Beta	*t* value	Lower *CI*	Upper *CI*	*p* value
Mini-BEST Total	0.03	0.48	0.61	0.47	1.50	0.49	0.04	0.41	Treatment	0.59	0.88	-0.74	1.92	*0.4*	-1.29	-2.09	-2.52	-0.07	***0.04***
									Order	0.23	0.35	-1.10	1.56	*0.7*	-0.32	-0.51	-1.54	0.91	*0.6*
									Period	0.35	0.52	-0.98	1.68	*0.6*	-1.36	-2.19	-2.59	-0.13	*0.03*
Mini-BEST APA	0.00	0.21	0.06	0.18	0.44	0.16	-0.42	0.14	Treatment	0.09	0.31	-0.46	0.63	*0.8*	-0.86	-4.01	-1.28	-0.43	***< 0.001***
									Order	-0.19	-0.68	-0.73	0.36	*0.5*	-0.02	-0.10	-0.44	0.40	*0.9*
									Period	0.31	1.15	-0.23	0.86	*0.3*	-0.06	-0.26	-0.48	0.37	*0.8*
Mini-BEST APR	0.13	0.20	0.30	0.26	0.06	0.20	0.25	0.19	Treatment	0.21	0.68	-0.41	0.84	*0.5*	0.20	0.73	-0.35	0.75	*0.5*
									Order	0.11	0.36	-0.51	0.74	*0.7*	-0.01	-0.05	-0.56	0.54	*1.0*
									Period	0.66	2.10	0.03	1.28	*0.04*	-0.10	-0.37	-0.65	0.45	*0.7*
Mini-BEST SO	-0.10	0.08	-0.03	0.11	0.00	0.14	0.06	0.13	Treatment	0.05	0.38	-0.22	0.32	*0.7*	0.11	0.57	-0.27	0.49	*0.6*
									Order	0.13	0.93	-0.15	0.40	*0.4*	-0.06	-0.30	-0.43	0.32	*0.8*
									Period	-0.18	-1.36	-0.46	0.09	*0.2*	-0.42	-2.24	-0.80	-0.05	*0.03*
Mini-BEST Gait†	0.00	0.30	0.27	0.24	1.00	0.25	0.15	0.26	Treatment	0.24	0.65	-0.50	0.98	*0.5*	-0.75	-2.17	-1.44	-0.06	*0.03*
									Order	0.18	0.49	-0.56	0.93	*0.6*	-0.23	-0.66	-0.91	0.46	*0.5*
									Period	-0.44	-1.18	-1.18	0.30	*0.2*	-0.77	-2.24	-1.46	-0.09	*0.03*
DTC Gait speed (%)	4.59	1.49	-1.25	1.10	5.30	1.50	1.29	1.51	Treatment	-5.60	-3.16	-9.16	-2.05	***0.003***	-3.78	-1.80	-7.97	0.40	*0.08*
									Order	0.98	0.55	-2.57	4.54	*0.6*	1.13	0.54	-3.04	5.31	*0.6*
									Period	2.72	1.53	-0.83	6.28	*0.1*	-2.05	-0.97	-6.23	2.13	*0.3*
DTC Stride length (%)	2.06	0.77	-0.48	0.71	3.33	1.26	1.72	1.53	Treatment	-2.37	-2.40	-4.34	-0.39	***0.02***	-1.43	-0.73	-5.34	2.47	*0.5*
									Order	1.06	1.08	-0.91	3.04	*0.3*	1.79	0.91	-2.11	5.68	*0.4*
									Period	2.12	2.15	0.15	4.10	*0.04*	-1.76	-0.90	-5.66	2.14	*0.4*
DTC_{cog} (%)	2.23	2.48	-5.04	2.33	1.33	2.48	5.08	4.21	Treatment	7.32	2.20	0.64	13.99	***0.03***	-4.81	-1.02	-14.15	4.52	*0.3*
									Order	-1.30	-0.39	-7.97	5.37	*0.7*	-3.96	-0.84	-13.29	5.37	*0.4*
									Period	-2.45	-0.74	-9.12	4.23	*0.5*	11.61	2.47	2.28	20.95	*0.02*
MDS-UPDRS Total	-3.94	1.70	1.97	1.79	-1.42	1.55	-0.98	1.98	Treatment	6.03	2.50	1.21	10.86	***0.02***	0.45	0.18	-4.56	5.46	*0.9*
									Order	-2.82	-1.17	-7.65	2.00	*0.2*	-0.51	-0.20	-5.52	4.50	*0.8*
									Period	0.62	0.26	-4.20	5.45	*0.8*	-0.01	0.00	-5.02	5.00	*1.0*
MDS_UPDRS Part II	-1.94	0.55	0.72	0.58	-0.62	0.51	-0.11	0.51	Treatment	2.68	3.41	1.11	4.25	***0.001***	0.37	0.52	-1.03	1.76	*0.6*
									Order	0.11	0.14	-1.46	1.68	*0.9*	0.44	0.62	-0.96	1.83	*0.5*
									Period	0.76	0.97	-0.81	2.34	*0.3*	1.32	1.87	-0.08	2.71	*0.07*

MDS-UPDRS Part III	-0.74	1.20	1.52	1.17	-1.90	1.06	-1.63	1.21	Treatment	2.21	1.36	-1.03	5.44	*0.2*	0.27	0.17	-2.92	3.46	*0.9*
									Order	-2.08	-1.29	-5.32	1.15	*0.2*	-0.32	-0.20	-3.50	2.86	*0.8*
									Period	-1.81	-1.12	-5.04	1.43	*0.3*	0.01	0.00	-3.18	3.19	*1.0*
PIGD score	-0.65	0.30	-0.27	0.33	-1.10	0.29	-0.15	0.28	Treatment	0.43	1.01	-0.42	1.28	*0.3*	0.96	2.41	0.17	1.75	***0.02***
									Order	-0.66	-1.56	-1.51	0.19	*0.1*	-0.24	-0.61	-1.03	0.55	*0.5*
									Period	0.57	1.35	-0.28	1.42	*0.2*	-0.09	-0.23	-0.88	0.70	*0.8*
PDQ-39 total	-2.64	0.85	0.55	0.92	-0.64	0.95	-0.63	0.67	Treatment	3.24	2.68	0.82	5.67	***0.01***	-0.21	-0.19	-2.50	2.07	*0.9*
									Order	-2.04	-1.68	-4.47	0.39	*0.1*	1.89	1.65	-0.39	4.18	*0.1*
									Period	0.53	0.43	-1.90	2.95	*0.7*	1.72	1.50	-0.56	4.00	*0.1*
PDQ-39 Mobility	-1.72	1.21	0.63	1.37	-0.09	1.34	1.19	1.14	Treatment	2.50	1.41	-1.05	6.04	*0.2*	1.11	0.64	-2.35	4.57	*0.5*
									Order	-1.17	-0.66	-4.72	2.38	*0.5*	2.06	1.17	-1.45	5.56	*0.2*
									Period	2.67	1.51	-0.88	6.22	*0.1*	1.20	0.69	-2.26	4.66	*0.5*
PDQ-39 ADL	-2.16	1.30	2.21	1.31	-1.46	1.21	1.94	1.22	Treatment	4.29	2.38	0.67	7.91	***0.02***	3.27	1.94	-0.08	6.62	*0.06*
									Order	-0.71	-0.39	-4.33	2.91	*0.7*	2.97	1.76	-0.38	6.31	*0.08*
									Period	-1.73	-0.96	-5.35	1.88	*0.3*	1.10	0.65	-2.25	4.44	*0.5*
SCOPA-COG	1.55	0.58	1.06	0.60	1.47	0.38	0.39	0.54	Treatment	-0.47	-0.57	-2.12	1.18	*0.6*	-1.01	-1.58	-2.28	0.26	*0.1*
									Order	-0.33	-0.40	-1.98	1.32	*0.7*	-0.20	-0.31	-1.46	1.07	*0.8*
									Period	-0.19	-0.23	-1.84	1.46	*0.8*	-1.15	-1.80	-2.42	0.12	*0.07*

Supplementary Table 3

The change of each clinical measure after exercise and education according to the baseline cognitive impairment

	No mild cognitive impairment (SCOPA-COG score ≥ 27)				Mild cognitive impairment (SCOPA-COG score < 27)														
	Delta Exercise		Delta Education		Delta Exercise		Delta Education		No mild cognitive impairment						Mild cognitive impairment				
Clinical measures	Mean	*SE*	Mean	*SE*	Mean	*SE*	Mean	*SE*	Fixed factor	Beta	*t* value	Lower *CI*	Upper *CI*	*p* value	Beta	*t* value	Lower *CI*	Upper *CI*	*p* value
Mini-BEST Total	0.67	0.39	0.55	0.34	1.50	0.75	-0.37	0.65	Treatment	-0.09	-0.18	-1.10	0.92	*0.9*	-1.80	-1.88	-3.73	0.13	*0.07*
									Order	-0.06	-0.12	-1.07	0.95	*0.9*	-0.34	-0.35	-2.27	1.59	*0.7*
									Period	-0.64	-1.25	-1.65	0.37	*0.2*	-1.05	-1.10	-2.98	0.88	*0.3*
**Mini-BEST APA**	0.07	0.14	-0.26	0.14	0.69	0.25	-0.19	0.19	Treatment	-0.35	-1.73	-0.74	0.05	*0.09*	-0.84	-2.86	-1.43	-0.25	***0.006***
									Order	-0.01	-0.05	-0.41	0.39	*1.0*	-0.23	-0.79	-0.82	0.36	*0.4*
									Period	0.35	1.75	-0.05	0.75	*0.08*	-0.59	-2.01	-1.18	0.00	*0.05*
Mini-BEST APR	0.20	0.17	0.31	0.17	-0.15	0.27	0.19	0.31	Treatment	0.11	0.45	-0.37	0.59	*0.7*	0.30	0.77	-0.49	1.09	*0.4*
									Order	0.06	0.26	-0.42	0.55	*0.8*	-0.03	-0.07	-0.82	0.76	*0.9*
									Period	-0.02	-0.07	-0.50	0.47	*0.9*	0.62	1.59	-0.17	1.42	*0.1*
**Mini-BEST SO**	-0.18	0.10	0.10	0.08	0.27	0.20	-0.15	0.22	Treatment	0.30	2.34	0.05	0.54	***0.02***	-0.38	-1.37	-0.95	0.18	*0.2*
									Order	-0.05	-0.37	-0.30	0.20	*0.7*	0.12	0.41	-0.45	0.68	*0.7*
									Period	-0.19	-1.52	-0.44	0.06	*0.1*	-0.62	-2.19	-1.18	-0.05	*0.03*
Mini-BEST Gait	0.58	0.23	0.40	0.21	0.69	0.38	-0.22	0.33	Treatment	-0.15	-0.49	-0.75	0.45	*0.6*	-0.88	-1.81	-1.87	0.10	*0.08*
									Order	-0.07	-0.22	-0.67	0.53	*0.8*	-0.19	-0.40	-1.17	0.79	*0.7*
									Period	-0.78	-2.58	-1.38	-0.18	*0.01*	-0.47	-0.97	-1.45	0.51	*0.3*
**DTC Gait speed (%)**	3.48	1.17	1.25	1.17	8.35	2.15	-2.02	1.92	Treatment	-2.26	-1.38	-5.51	0.98	*0.2*	-10.55	-3.74	-16.23	-4.87	***0.001***
									Order	1.27	0.77	-1.98	4.51	*0.4*	-0.16	-0.06	-5.84	5.51	*1.0*
									Period	-0.84	-0.51	-4.08	2.41	*0.6*	2.19	0.78	-3.49	7.87	*0.4*
**DTC Stride length (%)**	1.22	0.76	2.03	1.09	6.33	1.84	-1.94	1.87	Treatment	0.78	0.59	-1.83	3.39	*0.6*	-8.56	-3.39	-13.65	-3.46	***0.002***
									Order	1.75	1.33	-0.86	4.36	*0.2*	0.03	0.01	-5.06	5.13	*1.0*
									Period	-1.53	-1.16	-4.13	1.08	*0.2*	3.45	1.36	-1.65	8.54	*0.2*
**DTC_{cog} (%)**	1.96	1.52	-2.57	1.36	0.88	5.19	11.13	9.28	Treatment	4.40	2.22	0.46	8.34	***0.03***	-11.19	-1.09	-31.89	9.51	*0.3*
									Order	0.02	0.01	-3.92	3.96	*1.0*	-9.78	-0.96	-30.48	10.92	*0.3*
									Period	3.53	1.78	-0.41	7.47	*0.08*	13.09	1.28	-7.60	33.79	*0.2*
MDS-UPDRS Total	-3.44	1.20	-0.15	1.50	0.15	2.74	1.18	3.06	Treatment	3.26	1.72	-0.49	7.01	*0.09*	1.20	0.30	-6.98	9.38	*0.8*
									Order	-1.44	-0.76	-5.19	2.32	*0.4*	-1.47	-0.36	-9.65	6.71	*0.7*
									Period	0.70	0.37	-3.05	4.45	*0.7*	-1.83	-0.45	-10.01	6.35	*0.7*
**MDS-UPDRS Part II**	-1.25	0.41	0.56	0.41	-0.95	0.87	-0.52	0.83	Treatment	1.79	3.09	0.64	2.93	***0.003***	0.26	0.23	-2.01	2.54	*0.8*
									Order	0.22	0.39	-0.92	1.37	*0.7*	0.53	0.47	-1.74	2.80	*0.6*
									Period	0.48	0.83	-0.67	1.62	*0.4*	2.32	2.06	0.05	4.59	*0.046*
MDS-UPDRS Part III	-2.09	0.93	-0.28	1.01	-0.12	1.49	-0.70	1.75	Treatment	1.88	1.38	-0.82	4.57	*0.2*	-0.52	-0.23	-5.04	4.00	*0.8*
									Order	-1.59	-1.17	-4.28	1.11	*0.2*	0.84	0.37	-3.68	5.36	*0.7*
									Period	-0.42	-0.31	-3.12	2.28	*0.8*	-1.55	-0.69	-6.07	2.97	*0.5*
**PIGD score**	-0.93	0.22	-0.21	0.21	-0.92	0.48	-0.19	0.49	Treatment	0.75	2.51	0.16	1.34	***0.01***	0.68	1.03	-0.64	2.00	*0.3*
									Order	-0.60	-2.02	-1.19	-0.01	*0.05*	0.06	0.09	-1.26	1.38	*0.9*
									Period	-0.21	-0.70	-0.80	0.38	*0.5*	1.04	1.58	-0.28	2.36	*0.1*
**PDQ-39 Total**	-2.01	0.73	0.58	0.66	-0.22	1.39	-1.56	0.96	Treatment	2.52	2.60	0.59	4.44	***0.01***	-1.43	-0.89	-4.67	1.80	*0.4*

									Order	-0.87	-0.90	-2.80	1.05	*0.4*	2.66	1.65	-0.58	5.91	*0.1*
									Period	0.86	0.89	-1.06	2.79	*0.4*	1.58	0.98	-1.66	4.81	*0.3*
**PDQ-39 Mobility**	-1.38	0.88	1.95	0.85	0.60	2.23	-1.15	1.99	Treatment	3.20	2.67	0.82	5.58	***0.009***	-1.87	-0.65	-7.63	3.90	*0.5*
									Order	-1.03	-0.86	-3.41	1.35	*0.4*	3.97	1.39	-1.80	9.73	*0.2*
									Period	1.57	1.31	-0.81	3.95	*0.2*	2.09	0.73	-3.68	7.86	*0.5*
**PDQ-39 ADL**	-1.85	0.84	2.55	1.00	-1.50	2.11	1.04	1.80	Treatment	4.33	3.35	1.76	6.91	***0.001***	2.46	0.95	-2.74	7.65	*0.3*
									Order	-0.65	-0.50	-3.22	1.92	*0.6*	5.93	2.30	0.74	11.12	*0.03*
									Period	0.71	0.55	-1.86	3.28	*0.6*	-1.71	-0.66	-6.90	3.49	*0.5*
SCOPA-COG†	1.27	0.41	0.98	0.51	1.96	0.51	0.04	0.65	Treatment	-0.27	-0.43	-1.55	1.00	*0.7*	-1.87	-2.35	-3.46	-0.27	***0.02***
									Order	-0.15	-0.23	-1.42	1.13	*0.8*	-0.47	-0.59	-2.07	1.13	*0.6*
									Period	-0.66	-1.03	-1.94	0.61	*0.3*	-1.01	-1.27	-2.61	0.59	*0.2*
