# Supplementary material for: Effects of the agility boot camp with cognitive challenge (ABC-C) exercise program for Parkinson’s disease
Source: NPJ Parkinsons Dis. 2020 Nov 2;6:31. doi: 10.1038/s41531-020-00132-z (PMC7608677; doi:10.1038/s41531-020-00132-z)
Supplement: Supplementary file 2 — Reporting Summary [file 41531_2020_132_MOESM2_ESM.pdf]

nature research

Corresponding author(s): Martina Mancini

Reporting Summary

Nature Research wishes to improve the reproducibility of the work that we publish. This form provides structure for consistency and transparency in reporting. For further information on Nature Research policies, see Authors & Referees and the Editorial Policy Checklist.

Statistical parameters

When statistical analyses are reported, confirm that the following items are present in the relevant location (e.g. figure legend, table legend, main text, or Methods section).

n/a	Confirmed	
☐	☒	The exact sample size (*n*) for each experimental group/condition, given as a discrete number and unit of measurement
☐	☒	An indication of whether measurements were taken from distinct samples or whether the same sample was measured repeatedly
☐	☒	The statistical test(s) used AND whether they are one- or two-sided<br>*Only common tests should be described solely by name; describe more complex techniques in the Methods section.*
☐	☒	A description of all covariates tested
☐	☒	A description of any assumptions or corrections, such as tests of normality and adjustment for multiple comparisons
☐	☒	A full description of the statistics including central tendency (e.g. means) or other basic estimates (e.g. regression coefficient) AND variation (e.g. standard deviation) or associated estimates of uncertainty (e.g. confidence intervals)
☐	☒	For null hypothesis testing, the test statistic (e.g. *F*, *t*, *r*) with confidence intervals, effect sizes, degrees of freedom and *P* value noted<br>*Give P values as exact values whenever suitable.*
☒	☐	For Bayesian analysis, information on the choice of priors and Markov chain Monte Carlo settings
☒	☐	For hierarchical and complex designs, identification of the appropriate level for tests and full reporting of outcomes
☐	☒	Estimates of effect sizes (e.g. Cohen's *d*, Pearson's *r*), indicating how they were calculated
☐	☒	Clearly defined error bars<br>*State explicitly what error bars represent (e.g. SD, SE, CI)*

Our web collection on statistics for biologists may be useful.

Software and code

Policy information about availability of computer code

Data collection	MobilityLab v2
Data analysis	MATLAB R2018b, SPSS Statistics version 25.0

For manuscripts utilizing custom algorithms or software that are central to the research but not yet described in published literature, software must be made available to editors/reviewers upon request. We strongly encourage code deposition in a community repository (e.g. GitHub). See the Nature Research guidelines for submitting code & software for further information.

Data

Policy information about availability of data

All manuscripts must include a data availability statement. This statement should provide the following information, where applicable:

- Accession codes, unique identifiers, or web links for publicly available datasets
- A list of figures that have associated raw data
- A description of any restrictions on data availability

The data sets generated during the current study are available from the corresponding author on a reasonable request.

Field-specific reporting

Please select the best fit for your research. If you are not sure, read the appropriate sections before making your selection.

☒ Life sciences ☐ Behavioural & social sciences ☐ Ecological, evolutionary & environmental sciences

For a reference copy of the document with all sections, see nature.com/authors/policies/ReportingSummary-flat.pdf

Life sciences study design

All studies must disclose on these points even when the disclosure is negative.

Sample size	Sample size was originally computed for 2 groups of people including 60 people with PD and 60 with Frontal Gait disorders, however, the trial later changed to examine people with PD only. Power to detect differences between the intervention arms in a crossover trial via analysis of variance with level of significance set to 0.05 (SAS software v9.3). We assumed baseline levels and patterns of change similar to those reported in King 2013.
Data exclusions	No data were excluded
Replication	This is a clinical trial, we did not have resources to verify replication, but we made sure the outcome measures included have good test-retest reliability (from previous studies)
Randomization	Subjects were randomly assigned to either the exercise-first or education-first intervention by a computerized block randomization centrally held in the Research Electronic Data Capture (REDCap) database-scheduling mode. Randomization was implemented by an independent statistician using a block size of 12 subjects (6 in each intervention).
Blinding	The researchers who performed all baseline, midpoint and final tests remained blinded to group assignment throughout the duration of the study. The participants were blinded to our hypothesis and expected outcomes.

Reporting for specific materials, systems and methods

Materials & experimental systems

n/a	Involved in the study	
☒	☐	Unique biological materials
☒	☐	Antibodies
☒	☐	Eukaryotic cell lines
☒	☐	Palaeontology
☒	☐	Animals and other organisms
☐	☒	Human research participants

Methods

n/a	Involved in the study	
☒	☐	ChIP-seq
☒	☐	Flow cytometry
☒	☐	MRI-based neuroimaging

Human research participants

Policy information about studies involving human research participants

Population characteristics	Subjects with idiopathic Parkinson's disease were eligible if they were: (a) 50 to 90 years old; (b) on stable anti-parkinsonian medication; (c) able to stand or walk for 2 minutes without an assistive device; and (d) able to consent to participate and to follow testing and intervention procedures. Exclusion criteria for participation were: (a) comorbidities that contraindicates exercise participation, (b) significant musculoskeletal or peripheral nervous system disorders affecting balance, (c) excessive use of alcohol or recreational drugs, (d) deep brain stimulation surgery, and (e) contraindications to MRI scans
Recruitment	Patients were referred from the Parkinson Center of Oregon, fliers were distributed in the community (gym, support groups). We don't see any bias in recruitment, except considered participants were from only from Oregon.
